# Evaluation of Sublingual Varices Prevalence and Its Respective Factors in Two Iranian Nursing Homes in 2019

**DOI:** 10.22038/IJORL.2022.63364.3170

**Published:** 2022-07

**Authors:** Aryan Jafari, Arezoo Alaee, Massoud Rezai, Mahdi Masoudi

**Affiliations:** 1 *Dental Material Research Center, Dentistry Faculty, Islamic Azad University of Medical Sciences, Tehran, Iran.*

**Keywords:** Age factors, Cross-sectional study, Hypertension, Dentures, Sublingual varices, Oral health, Preventive health services, Smoking, Geriatrics

## Abstract

**Introduction:**

Sublingual varices (SLVs) are among the most prevalent oral lesions, which develop with aging. We aimed to find the prevalence of SLVs among seniors in two nursing homes and evaluate the possible linked factors.

**Materials and Methods:**

This descriptive cross-sectional study was carried out at Kahrizak Alborz and razy allah razi Al-Waledain nursing homes in 2019. The list of all seniors over 60 years old was prepared then; after explaining the aim of the study and obtaining their consent, a well-trained senior dentistry student examined them for the presence of SLVs. At the same time, factors, including age, gender, smoking, oral prosthesis, leg varices, high blood pressure, and literacy level, were recorded. The role of each feature was analyzed by Chi-square test using SPSS (version 22; SPSS Inc., Chicago, IL, USA).

**Results:**

The study performed on 478 nursing home residents showed an SLVs' prevalence of 56.7% (95% confidence interval (CI): 52.3-60). SLVs were significantly correlated with gender (P<0.001), age P<0.01), smoking status (P<0.001), complete denture usage (P<0.01), and leg varicosity status (P<0.0001).

**Conclusions:**

It appears that SLVs are highly prevalent in senior adults. Therefore, clinicians should be aware of the possible presence of SLVs and avoid unnecessary interventions.

## Introduction

Sublingual varices (SLVs), also known as tongue varicosities, oral phlebectasia linguae, lingual varicosities, and caviar tongue, are a relatively common typical finding, especially among the seniors. Oral varices are believed to be benign evolutionary anomalies. Varicose is irregularly twisted and widened veins, most of which are located in the mouth on the abdominal surface of the tongue. They are most commonly visible on the tongue's ventral side, characterized by several small, irregular, blue/purple lesions with a bilateral linear distribution from the back of the tongue to the tip (1). However, a recent study documented a rare occurrence of a massive thrombosed SLV with a diameter of 2 centimeters (2). They can also appear on the lips and mouth floor. The labial commissures, buccal mucosa, and occasionally the soft and hard palate occur less commonly (3). Their pathophysiology is unknown; however, it may be attributed to alterations in connective tissue or the aging-associated venous walls' deterioration due to degeneration of elastic fibers (4). 

These lesions have an unsightly look and can create anxiety and dread in those who notice their presence in their mouth; in definite cases, a person may develop a cancer phobia, which necessitates sampling and conducting a dental para clinic examination. In most cases, SLVs require no therapy other than reassurance about their benign nature. Infrequently, patients on anticoagulants and antiplatelet treatment may have a spontaneous or trauma-related hemorrhage. Therefore, surgical or laser-assisted removal of the varix is suggested as a precaution occasionally in such instances (5–7). 

SLVs are often asymptomatic and mainly detected during oral examinations. SLV would be soft, compactable, frequently painless, and white at the touch via a diascopy test (8). 

SLVs demonstrate blanching during diascopy, proving their vascular origin. It must also be distinguished from hemangiomas, lymphangiomas, melanoma, Kaposi sarcoma, and blue rubber bleb nevus, which may be done by collecting a thorough medical history and performing a detailed clinical examination. These points are highly noteworthy in differentiating SLVs from melanotic or purpuric lesions that do not change coloration with pressure (1). SLVs have been linked to hypertension, cardiovascular disease, smoking, hepatic cirrhosis, tooth attrition, denture usage, and leg varicosity (9–12). In contrast to varix lesions of the lower limbs, which are substantially more prevalent in women (13), a survey with 1751 samples found that SLVs develop sooner and slightly tend in males across all age groups (14).

The population age distribution in developed and developing countries is expected to change dramatically by 2050. The world's population is aging which is the most significant demographic change. According to the UN, people over 80 years old will make up roughly 20% of the global population in the future (15). As a result, the senior population will soon account for a significant share of dental care customers. Atrophic glossitis, SLVs, and fissured tongue are oral lesions that develop as people age (16–18). Studies have estimated SLVs prevalence at approximately 22.5 percent, prevalent in those over the age of 60 and rare in youngsters (5,8). A young individual's vascular lesion on the tongue can indicate a congenital condition like Osler or Fabry syndrome (4). Thus, clinicians can benefit from understanding common oral conditions in older adults. It will save patients unnecessary therapies and surgeries.

A study among institutionalized senior individuals in Mashhad, Iran, suggested that SLV (42%) is the third most prevalent oral lesion after fissured tongue (66.5%) and atrophic glossitis (46.8%) (19). However, the most common oral lesion, according to Rabiei, is dry mouth (42.1%), followed by the fissured tongue (29.9%), atrophic glossitis (25.9%), and SLV (22.7%) (20).

The present study was conducted on the senior individuals of two nursing homes to address these inconsistencies between various studies regarding SLVs' prevalence and the associated factors.

## Materials and Methods

The protocol for this observational, cross-sectional study followed the Helsinki declaration and was approved by Tehran Islamic Azad University of medical sciences' ethical committee (IR.IAU. DENTAL.REC.1399. 134). 

Of the 509 residents of these two nursing homes, thirty-one individuals could not participate in the study and were dropped due to certain conditions (e.g., dementia, paralysis, etc.). Hence, the research was carried out using census sampling among 478 individuals over 60 years old in two nursing homes, Kahrizak Alborz and razy allah razi Al-Waledain. Informed consent was obtained from every individual. The patients were instructed to move their tongue upward, then left and right to check the tongue's ventral surface and lateral borders. A senior dentistry student trained by an oral medicine specialist to diagnose SLVs, examined the individuals using a dental mirror, explorer, cotton roll, cotton pliers, and flashlight as the light source. The presence or absence of SLV and patients' characteristics, including age, gender, literacy level, smoking (who smoked at least one cigarette daily and over the six months (21)), complete or partial removable dentures, and leg varices were recorded. The blood pressure average, acquired from the participant's daily medical check-up report throughout a week, was used to measure their blood pressure. While 120/80 mmHg is considered normal blood pressure, hypertension was defined as a blood pressure reading of 140/90 mmHg or above (22).

Statistical analysis was performed using the Chi-square test using SPSS software (version 22; SPSS Inc., Chicago, IL, USA). A p-value of less than 0.05 was considered statistically significant.

## Results

A total of 271 (56.7%) individuals had SLV (95% confidence interval (CI): 52.3-60), of which 176 had bilateral SLVs. 

The study was conducted on 478 individuals from 509 residents of two nursing homes, including 202 (42.3%) male and 276 (57.7%) female. While 11.9% were smokers, 81% had more than two years of residence in nursing homes. As Table 1 demonstrates the prevalence of SLV and the association with different factors, SLVs occurrence was correlated with gender (P< 0.001), smoking status (P< 0.001), age (P<0.01), complete denture use (P<0.01) and leg varicosity status (P<0.0001). 

However, SLVs’ presence was not significantly correlated with blood pressure (P=0.8), level of literacy (P=0.8), length of stay (P=0.09), or removable partial denture usage (P=0.9).

**Table 1:** Prevalence of sublingual varices (SLVs) and the associated factors

**Associated factors **		**Without SLV** **N**_1_ **= 207**	**With SLV** **N**_2_ **=271**	**P value**
Gender	Male Female	67 (22.4%) 140 (67.6%)	135 (49.8%) 136 (50.2%)	<0.001
Smoking	Smoker Nonsmoker	10 (4.9%) 197 (95.1%)	47 (17.4%) 224 (82.6%)	<0.001
Length of stay **(Mean: 2 years)**	< 2 years ≥ 2 years	169 (81.7%) 38 (18.3%)	218 (80.4%) 53 (19.6%)	0.9
Age **(Mean:74.5 years)**	≥74.5 years <74.5 years	164 (79.2%) 43 (20.8%)	136 (50.2%) 135 (49.8%)	<0.01
Blood pressure	≥140/90 mmHg 120/80-140/90 mmHg <120/80 mmHg	74 (35.8%) 136 (51.2%) 27 (13%)	103 (38%) 164 (60.5%) 4 (1.5%)	0.8
Complete denture	Have Do not have	49 (24%) 158 (76%)	141 (52%) 130 (48%)	<0.01
Removable partial denture	Have Do not have	19 (9%) 166 (91%)	22 (8%) 249 (92%)	0.9
Leg varices	Have Do not have	73 (35.3%) 134 (64.7%)	138 (51%) 133 (49%)	<0.0001
Literacy	Diploma or higher Lower than diploma	45 (21.8%) 162 (78.2%)	48 (17.8%) 223 (82.2%)	0.8

## Discussion

Our research showed that the prevalence of SLVs among seniors was 56.7%. Prevalence is the proportion of the population with a given condition or disease (22). The oral lesions prevalence was 34.8% reported by Owlia et al. This report was a case of all types of oral lesions and did not mention SLV's prevalence separately (23). Also, according to Lynge Pedersen et al., SLVs are the most common oral lesion in older adults, with a 28.3% prevalence. Moreover, SLVs were more common among the senior individuals with systemic disorders and drug use, mainly cardiovascular disease (24).

Molania et al. reported an almost 100% prevalence of oral lesions in the aged people in a cross-sectional study of 90 individuals living in nursing homes in northern Iran, of which 21.1% were SLVs (25). The reported prevalence of SLV in their survey is lower than in the present study. Its possible explanation can be the lack of cooperation of some older people, a smaller sample size, or more favorable living conditions in the nursing homes of northern Iran.

Hedström et al. reported SLVs prevalence to be 72.7% (10). These disparities might be related to the research samples' varying ages. Furthermore, the diagnostic criteria, methodology, and sample methodologies used in various research may differ.

In another study by Hedström et al., age, hypertension, and smoking were significantly correlated with SLV, conforming to our research (4); though, emphasizes the lack of a relationship between the gender of people with SLVs, inconsonant with our findings indicating significant relationship between gender and SLVs. This inconsistency may be due to cultural differences, smoking, or diet dissimilarity. Demirel et al. claimed a significant relationship between SLVs and hypertension, which contradicts our findings. However, an epidemiological study cannot match the limited sample size (26). Al-Shayyab et al. reported the SLV's prevalence to be 20.5%, while 34% were unaware of the lesion's presence (5). The prevalence rate reported by Al-Shayyab et al. is much lower than our results since the subjects studied by Al-Shayyab et al. were patients who attended a hospital's dentistry department and were younger than our samples (the seniors in two nursing homes). Indeed, younger adults are usually more careful and obsessive regarding their oral health than older ones. As previously stated by others (5,12), using removable prostheses was strongly associated with SLV's presence. Nevertheless, the length of time that dentures were worn, was not taken into account in our study like theirs. 

However, they stressed the need to wear dentures, particularly for an extended period in oral disorders, including denture hyperplasia and denture stomatitis. Poor oral hygiene and inflammation created by using a prosthesis were thought to be the causes of these denture-induced lesions.

Akkaya et al. found out that gender, systemic disorders, smoking, and denture wear are variables linked to SLVs after conducting a study on 691 patients (470 females, 221 males) who visited Hacettepe University's Department of Dentomaxillofacial Radiology. They also claimed that ex-smokers had a higher prevalence of SLVs than current smokers. This finding may be linked to nicotine's vasoconstrictive properties, which may help inhibit the formation of varicose veins (3). A case-control study was done on 222 persons over 65 at a nursing home by Barzideh et al. Like our study, older people with SLVs are more prone to consume cigarettes (8).

Oral health safety in nursing homes, especially for elderlies, is of utmost importance; it must be carefully done as authorized screening episodes by trained clinicians. Dental faculties, dental students, dentists, or non-governmental organizations (NGOs) should participate in such programs. It could increase the health score in society and prevent the oral pre-cancerous and cancerous lesions and malpractice trend (27). 

Cooperation between welfare organizations for disabled people and educational planning centers to visit and screen the nursing home residents can have spiritual effects on visitors' lives and diagnostic aims. 

Though, the present study had some limitations, which may have caused some shortcomings. Finding nursing homes eager to cooperate had some difficulties. The limited-time to examine samples, which began after breakfast and ended before lunch, caused some problems. Considering that seniors were eager to talk and had a good feeling, it was difficult and disrespectful to interrupt their talking and proceed to examine another individual. There was no bias in this study. 

All individuals were examined for SLVs, and an oral medicine specialist monitored suspicious lesions.

## Conclusions

It is determined that SLVs are highly prevalent in older people. It is recommended to investigate the prevalence of SLVs in the Iranian population with a larger sample size in a multi-centered study.
